# The impact of a multidisciplinary cardio‐oncology programme on cardiovascular outcomes in Taiwan

**DOI:** 10.1002/ehf2.12840

**Published:** 2020-07-04

**Authors:** Wei‐Ting Chang, Yin‐Hsun Feng, Yu Hsuan Kuo, Wei‐Yu Chen, Hong‐Chang Wu, Chien‐Tai Huang, Wen‐Ching Wang, Chia‐Te Liao, Zhih‐Cherng Chen

**Affiliations:** ^1^ Division of Cardiology, Department of Internal Medicine Chi‐Mei Medical Center 901, Zhonghua Road Tainan Taiwan; ^2^ Department of Biotechnology Southern Taiwan University of Science and Technology Tainan Taiwan; ^3^ Institute of Clinical Medicine, College of Medicine National Cheng Kung University Tainan Taiwan; ^4^ Division of Oncology, Department of Internal Medicine Chi‐Mei Medical Center Tainan Taiwan; ^5^ Division of General Surgery, Department of Surgery Chi‐Mei Medical Center Tainan Taiwan

## Introduction

Anthracycline toxicity is well known.^1^ Early detection of minor myocardial dysfunction is important for the prevention of subsequent cardiotoxicity.^1^ Cardio‐oncology is a multidisciplinary field that focuses on management and prevention of cardiovascular complications in cancer patients.^2^ A recent study in the United Kingdom showed that a cardio‐oncology programme led to higher rates of cancer treatment continuation and cardiac optimization.^3^ However, it remains largely unknown whether a cardio‐oncology programme actually makes a difference in cardiovascular outcomes. From another perspective, contrary to early awareness of the importance of cardio‐oncology programmes in Western countries, Asian countries did not pay much attention to this issue until recently. Although the Korean Society of Echocardiography recently published an opinion paper focusing on cardio‐oncology, the incidence of heart failure (HF) and cardiovascular outcomes requires further research.^4^

### Objective

This study aimed to investigate the incidence of chemotherapy‐induced myocardial dysfunction in breast cancer patients preparing for anthracycline therapy and whether there is a beneficial effect of multidisciplinary team in cardio‐oncology care.

## Methods

This prospective study shares our 4 year experiences at Chi‐Mei Medical Center, Taiwan, establishing a cardio‐oncology programme and its impact on the clinical outcomes of breast cancer patients undergoing anthracycline therapy. Newly diagnosed breast cancer patients preparing for anthracycline therapy were enrolled from 2014 to 2018. After excluding three subjects with underlying HF, five with poor echo imaging and three who refused to attend the programme, a total of 154 patients were finally enrolled. Sequential echocardiography was performed at baseline, 3 months, 6 months, and 1 year. With a 3.5 MHz multiphase‐array probe (Vivid E9; GE Vingmed Ultrasound AS, Horten, Norway) in accordance with recommendations of the American Society of Echocardiography, left ventricular ejection fraction (LVEF) was measured using biplane Simpsons method. Although not shown in this study, speckle‐tracking echocardiography has also been analysed for myocardial deformation. According to the most commonly used definition, cancer therapy‐related cardiac dysfunction (CTRCD) is defined as a significant drop in the LVEF of more than 10% to below 50%. Additionally, biomarkers including high‐sensitivity troponin I and N‐terminal pro‐brain natriuretic peptide, 6 min walking distance and adverse cardiovascular events including new‐onset hypertension, HF, ischemic stroke, enzyme‐positive myocardial infarction and all‐cause mortality were recorded at each visit. Quality of life questionnaires including the EuroQol‐5D (EQ 5D) index and EQ 5D visual analogue scale were administered before, during, and after anti‐cancer therapies. In this cardio‐oncology programme, any functional decline was reported to oncologists to consider adjustment of regimens. Otherwise, cardiologists were consulted for education and management of cardiovascular risks, including hypertension, diabetes, and hyperlipidaemia. For patients with either cardiovascular risks or asymptomatic LVEF reductions, medications including anti‐platelets/anti‐coagulants, angiotensin‐converting enzyme inhibitors/angiotensin receptor blockers, β‐blockers, mineralocorticoid receptor antagonist, and statins were prescribed upon the clinical judgement of the cardiologists.

Echocardiographic and clinical records of 450 breast cancer patients undergoing anthracycline therapy from 2010 to 2013, before the cardio‐oncology programme, were also collected as a comparison (non‐cardio‐oncology programme). The selection of the comparison group was based on the same anti‐cancer regimens used. The median follow‐up duration was 30 months (22–38 months) for both groups. The study was conducted in strict accordance with the Declaration of Helsinki and was approved by the local ethics committee (institutional review board approval 10411‐008).

### Statistic

Continuous data were expressed as means ± standard deviations, whereas dichotomous data were expressed as numbers and percentages. Chi‐square tests or Fisher's exact tests were used for categorical variables. Student's *t*‐tests and nonparametric tests were used for comparison depending on the distribution of continuous variables. SPSS software (Version 22.0, IBM SPSS Inc., Chicago, IL, USA) was applied for statistical analyses.

## Results

**Figure 1:**
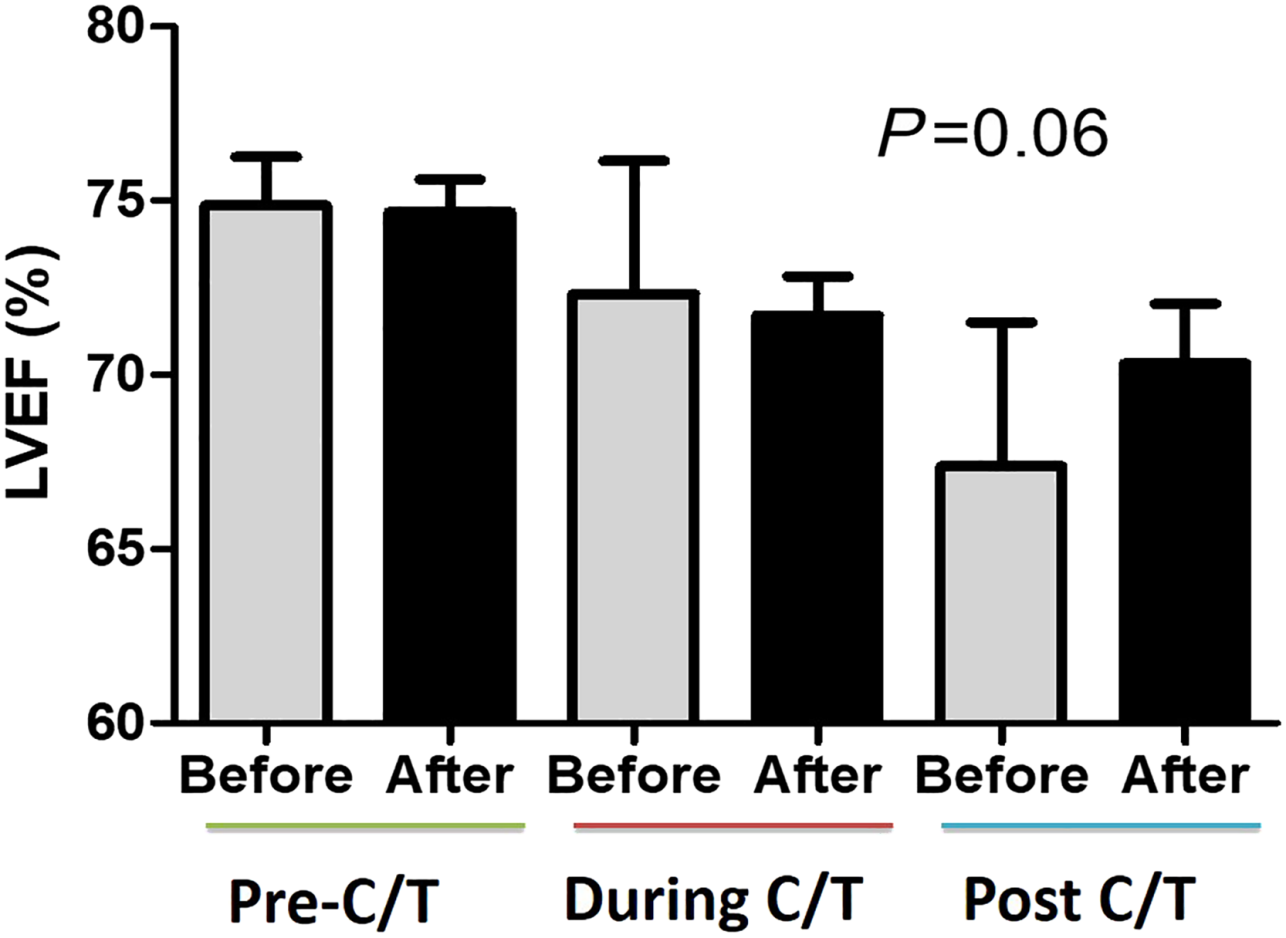
In contrast to a continuous drop of left ventricular ejection fraction (LVEF) in the non‐cardio‐oncology programme, the decline of LVEF was mitigated in patients under the cardio‐oncology programme. (Grey bar: non‐cardio‐oncology programme, *N* = 154; black bar: cardio‐oncology programme, *N* = 450).

Ages between non‐cardio‐oncology and cardio‐oncology programme groups, (53.8 ± 11.5 vs. 53.7 ± 10.1 years old; *P* = 0.87) were similar (*Table* *1*). Likewise, there was no significant difference in the prevalence of background cardiovascular risks such as hypertension (7.1% vs. 6.5%; *P* = 0.52), diabetes (2.2% vs. 1.9%; *P* = 0.43), hyperlipidaemia (6.2% vs. 5.2%; *P* = 0.21), and chronic kidney disease (1.3% vs. 1.3%; *P* = 0.68) between the two groups. None of the patients smoked. The majority of patients were diagnosed at Stage 2 breast cancer. Furthermore, the average single and accumulating doses of anthracycline use were similar (non‐cardio‐oncology vs. cardio‐oncology programme: 119.7 ± 12.9 vs. 119.1 ± 20.8 mg/M^2^, *P* = 0.61; 313.7 ± 108.9 vs. 309.17 ± 82.9 mg/M^2^, *P* = 0.92, respectively). The percentage of trastuzumab use or concomitant radiotherapy was approximately 25% in both groups. Only a small portion of patients received cardiovascular medications for hypertension or primary prevention. Among all enrolled patients, 20 (12.5%) of them were referred to a cardiologist for cardiovascular risk management while none discontinued anthracycline therapy. In the non‐cardio‐oncology programme, the ratios of echocardiography screening were only 36%, 18%, and 10% before, during, and after anthracycline therapy, respectively. Conversely, in the post‐cardio‐oncology programme, the ratios of echocardiography were each close to 100%. Of note, among the patients in the non‐cardio‐oncology program, LVEF continuously dropped as the doses of anthracycline accumulated (before, during, and after: 74.8 ± 8.6%, 72.3 ± 12%, and 67.3 ± 10.1%, respectively). Conversely, after the cardio‐oncology programme, the insignificant trend in the LVEF decline was mitigated (74.6 ± 7.2%, 71.7 ± 8.3%, and 70.3 ± 8.1%, respectively) (*Figure* *1*).

**Table 1:** The baseline characteristics of the breast cancer patients under cardio‐oncology programme or not

Characteristic	Non‐cardio‐oncology programme (*n* = 450)	Cardio‐oncology programme (*n* = 154)	*P* value
Demographic parameters			
Age (years)	53.8 ± 11.5	53.7 ± 10.1	0.87
Body height (cm)	155.0 ± 13.5	156.5 ± 5.8	0.47
Body weight (kg)	60.6 ± 12.8	58.3 ± 6.3	0.62
Heart rate (bpm)	80.6 ± 10.1	80.5 ± 11.5	0.64
DM, *n* (%)	10 (2.2)	3 (1.9)	0. 43
HTN, *n* (%)	32 (7.1)	10 (6.5)	0.52
Hyperlipidaemia, *n* (%)	28 (6.2)	8 (5.2)	0.21
CKD, *n* (%)	6 (1.3)	2 (1.3)	0.68
Laboratory parameters			
Creatinine clearance rate (mL/min)	83.7 ± 34.5	95.7 ± 45.8	0.29
Serum glucose (ac, mg/dL)	95 ± 8.5	98.7 ± 18.9	0.11
Triglyceride (mg/dL)	129.0 ± 6.9	129.5 ± 6.2	0.79
Cholesterol (mg/dL)	182.6 ± 30.6	174.6 ± 21.6	0.75
NT‐proBNP (pg/mL)	12.9 ± 12.0	10.2 ± 14.1	0.27
hsTnI (pg/mL)	2.6 ± 2.5	3.2 ± 2.11	0.25
Anti‐cancer therapies			
Cancer stage			
1	112 (24.8)	33 (21.4)	0.62
2	225 (50)	84 (54.5)	0.58
3	68 (15.1)	26 (16.8)	0.71
4	45 (10)	11 (7.1)	0.34
Operations, *n* (%)	291 (64.6)	85 (55.2)	0.12
Mean single dose of Anthracycline (mg/M^2^)	119.7 ± 12.9	119.1 ± 20.8	0.61
Accumulating dose of Anthracycline (mg/M^2^)	313.7 ± 108.9	309.17 ± 82.9	0.92
Trastuzumab use, *n* (%)	106 (23.5)	38 (24.7)	0.54
Concomitant radiotherapy, *n* (%)	108 (24)	46 (29.9)	0.12
CV medications			
Anti‐platelet/anti‐coagulants, *n* (%)	4 (0.8)	3 (1.9)	0.54
ACEIs/ARB, *n* (%)	23 (5.1)	10 (6.5)	0.82
MRA, *n* (%)	5 (1.1)	2 (1.3)	0.63
β‐blockers, *n* (%)	18 (4)	9 (5.8)	0.58
Statins, *n* (%)	13 (2.8)	5 (3.2)	0.6

ACEIs/ARB, angiotensin‐converting enzyme inhibitors/angiotensin receptor blocker; BNP = brain natriuretic peptide; CKD, chronic kidney disease; CV, cardiovascular; DM, diabetes mellitus; hsTnI, high*‐*sensitivity troponin I; HTN, hypertension; MRA, mineralocorticoid receptor antagonist; NT‐proBNP, N‐terminal pro‐brain natriuretic peptide.

Data are expressed as mean ± SD or median. Breast cancer staged is based on ‘AJCC staging systems’.

In this cohort, only five (3.2%) patients fulfilled the CTRCD criteria while none developed HF symptoms or cardiovascular complications with the cardio‐oncology programme (*Table* *2*). Among five patients with CTRCD, their average ages were young (48.2 ± 12.8), and only one had hypertension under treatment, whereas the other four patients were free from traditional cardiovascular risks. Of note, myocardial function of these five patients returned to baseline after lifestyle modifications, risk factor management and medical interventions (especially angiotensin‐converting enzyme inhibitors/angiotensin receptor blockers). Among them, none ceased or changed their anti‐cancer regimens while one postponed the anthracycline therapy for 3 months.

**Table 2:** The individual characteristics of the five breast cancer patients developing cancer therapy related cardiac dysfunction (CTRCD)

Patient number	Age (y/o)	Cancer stage	CV risk factors	Trastuzumab	Anthracycline doses (mg/M^2^)^a^	Radiotherapy	Baseline LVEF (%)	Cardiotoxicity LVEF (%)	CV Mx	Postponed anti‐cancer therapies	The followed LVEF (%)
1	45	2	None	+	200	−	70	49	Valsartan	None	60
2	55	3	None	−	420	−	63	48	Carvedilol	None	58
3	62	2	HTN	−	460	+	62	45	Acertil	3 months	59
4	35	3	None	+	280	+	65	47	Valsartan	None	65
5	33	2	None	−	440	−	58	45	Bisoprolol	None	62

CV, cardiovascular; HTN, hypertension; LVEF, lefty ventricular ejection fraction; Mx, medication

Breast cancer staged is based on ‘AJCC staging systems’.

^a^ Accumulating does of anthracycline before CTRCD.

**Figure 2:**
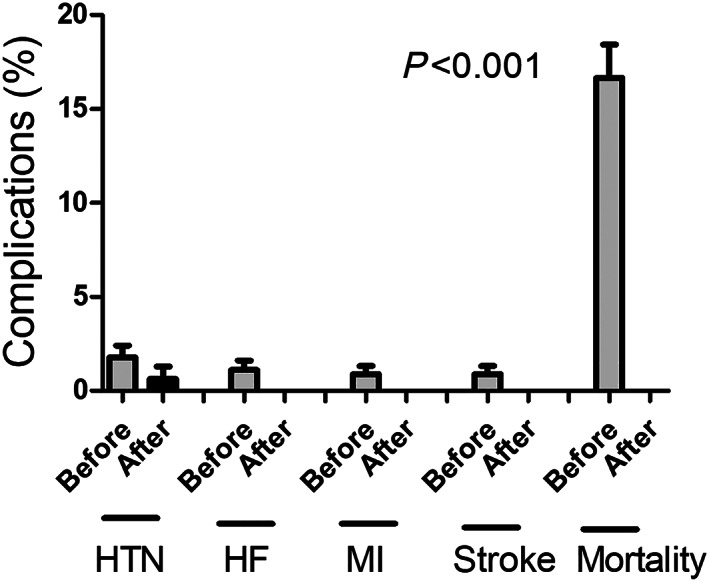
Before cardio‐oncology programme, there were 1.7% of new‐onset hypertension (HTN), 1% of new‐onset heart failure (HF), 0.9% of myocardial infarction (MI), 0.9% of ischemic stroke, and 16.8% of mortality. Conversely, after the programme, there were only 0.6% of new‐onset hypertension while no other cardiovascular complications were reported.

Compared with a 1% incidence of new‐onset HF in the non‐cardio‐oncology program, none of the patients in the cardio‐oncology programme developed HF symptoms during the follow‐up period. Before the cardio‐oncology programme, there was 1.7% of new‐onset hypertension, 1% of HF, 0.9% of myocardial infarction, 0.9% of ischemic stroke, and 16.6% of all‐cause mortality in patients undergoing anthracycline therapy. After the programme, there was only 0.6% of new‐onset hypertension while no other cardiovascular complications were reported (*Figure* *2*).

Regarding biomarkers, although there were immediate increases in high‐sensitivity troponin I and N‐terminal pro‐brain natriuretic peptide after anthracycline therapy, most gradually returned to baseline after completion of treatment. Similarly, although the 6 min walking distance declined slightly during the therapies (from 393.6 ± 119.8 to 371.4 ± 84.2 m), it improved thereafter. In the quality of life analysis, both EQ 5D index and visual analogue scale declined slightly (14.2 ± 0.5 and 76 ± 10.6) immediately after anthracycline therapy compared with baseline (14.6 ± 0.9 and 78.2 ± 13.2). Notably, they returned to normal and were even higher than at baseline (14.8 ± 0.4 and 79 ± 11.3) within a year among patients enrolled in the cardio‐oncology programme.

## Discussion

In this real‐world experience of the first prospective cardio‐oncology programme in Taiwan, we found that first, despite low cardiovascular risks at baseline, a percentage of patients continued to experience a decline in LVEF or even developed CTRCD after anthracycline therapy, and second, through either education or medical intervention, the early identification and management of the cardiovascular risks was associated with improvements of cardiovascular outcomes and quality of life. The reason for the different outcomes between the cardio‐oncology and non‐cardio‐oncology groups may attribute to the management of cardiovascular risks, early medical interventions for declined myocardial function, even asymptomatic, and lifestyle modification while more evidences are required. Similar to this study, some of the patients developing CTRCD were young and free from traditional cardiovascular risks.^5^ In previous studies, elevated serum brain natriuretic peptide and troponin levels may not have been sensitive enough to differentiate patients with and without cardiotoxicity at an immediate stage.^6^ Although combining advanced imaging including cardiac magnetic resonance imaging and speckle‐tracking echocardiography may provide a higher reliability of risk stratification,^4^, ^5^, ^7^ early and cost‐effective identification of at‐risk patients remains a major challenge. Prevention strategies for anti‐cancer therapy‐induced cardiotoxicity include prevention prior to treatment and monitoring the development of cardiotoxicity during and after treatment. Despite previous studies showing that the institution of HF treatments at baseline in high‐risk patients could reduce symptomatic HF and LVEF declines,^8^ the largest clinical trial of β‐blockers for the prevention of cardiotoxicity demonstrated no impact on the incidence of early‐onset LVEF reduction.^9^ Therefore, the sequential monitoring and early intervention of HF management is presently the most evident management for CTRCD.

### Limitations

This study has some limitations. First, the small number of patients diagnosed with CTRCD limited further statistical analysis, including logistic regression. Second, the non‐randomized study design may attenuate accuracy, especially in patients who received echo examinations already at high cardiovascular risks. With a retrospective approach, several parameters (e.g. quality of life) are missing in the control group. Third, given that aetiologies cannot be comprehensively identified, the all‐cause mortality could be cancer or cardiac related (*Figure* 2). Finally, despite the zero event rate reported for the cardio‐oncology group, it should be interpreted with caution, and long‐term follow‐up is required to differentiate the actual impact of cardio‐oncology care in the study population.

## Conclusions

In this first real‐world experience in Taiwan, we found that cardio‐oncology team care improved the recovery of myocardial function, mitigated adverse events, and maintained cancer patients within anti‐cancer therapies. Because many cancer survivors do not receive guideline‐directed HF therapies, a cardio‐oncology programme is an opportunity for collaboration between oncologists and cardiologists to improve the care of oncology patients and maintain them on anti‐cancer treatments. In spite of some limitations, this study emphasized the importance of comprehensive monitoring and early intervention of myocardial dysfunction after chemotherapy.

## Conflict of interest

None declared.

## Funding

This study was supported by Chi‐Mei Medical Center.
